# Case report: From oral infection to life-threatening pneumonia: clinical considerations in Nocardia infection from a case

**DOI:** 10.3389/fmed.2024.1424564

**Published:** 2024-07-17

**Authors:** Kang Chen, Ying Wang, Jing Dong, Ping-Shang Wu, Jun Yang, Guo-Ping Ai

**Affiliations:** ^1^Department of Thoracic Cardiovascular Surgery, The Third Hospital of Wuhan, Wuhan, China; ^2^Department of Otolaryngology, Qingdao Hospital, University of Health and Rehabilitation Sciences (Qingdao Municipal Hospital), Qingdao, China; ^3^Department of Endocrinology, General Hospital of Central Theater Command of the People's Liberation Army, Wuhan, China; ^4^Department of Stomatology, General Hospital of Central Theater Command of the People's Liberation Army, Wuhan, China; ^5^Department of Radiology, General Hospital of Central Theater Command of the People's Liberation Army, Wuhan, China

**Keywords:** Nocardia, severe acute diseaseintensive care, immunocompetent patient, pulmonary coinfection, case report

## Abstract

Nocardia is an anthropozoonotic bacteria that occurs widely in the natural environment. However, because it is a gram-positive aerobic opportunistic pathogen, it rarely occurs in patients with no prior history of immune function disease. Since the symptoms are nonspecific the diagnosis of Nocardia pneumonia is challenging. Previous studies have not reported that this anthropozoonotic bacteria colonizing the human body could cause severe pneumonia by gingival pain and pharyngeal discomfort. This case report describes a previously healthy 60-year-old female farmer who presented to the doctor with gingival pain and pharyngeal discomfort. She was treated with a dental cleaning and oral metronidazole. The patient rapidly progressed to breathing difficulties. Lung shadow was found by computerized tomography examination. The radiologist diagnosed pulmonary tuberculosis as image-based. Through laboratory examination and culture of pathogenic microorganisms in the sputum and blood of the patient, no obvious positive findings were found. The disease progressed rapidly to tracheal intubation ventilator assisted breathing. Subsequently, the patient underwent alveolar lavatory examination under endotracheal intubation fiberbronchoscopy, and the culture of alveolar lavage fluid indicated Nocardia. According to this result, the patient’s disease was quickly controlled after selecting the targeted drug compound sulfamethoxazole and intravenous meropenem for treatment. In view of the reason for the high misdiagnosis rate due to the low positive rate of Nocardia culture in most cases, the clinical thinking of diagnosis and treatment from oral infection symptoms to fatal pneumonia reported in this case has certain clinical popularization and enlighten significance, not only improved the diagnosis and treatment of rare diseases, but also be reduced medical disputes.

## Introduction

The clinical manifestations and imaging examinations of pulmonary nocardiosis were nonspecific (1). In addition to the detection of pathogens by culture or next-generation sequencing (NGS) analysis of species, source, and drug sensitivity of pathogens, general laboratory tests also had no specificity and/or sensitivity indicators (2, 3). However, Nocardia pneumophila grew very slowly in *in vitro* medium and was easily covered by other fast-growing bacteria, which made it difficult to be detected (4, 5). Nocardia, as an opportunistic anthropozoonotic bacteria pathogen, is resistant to general antibiotics (6). As a result, infection of the lungs of even healthy people with this organism can lead to rapid progression and catastrophic consequences (7). We report a case of pulmonary nocardiosis with rapid progression from oral symptoms in order to promote the attention of clinicians to this disease.

## Case report

**Figure 1:**
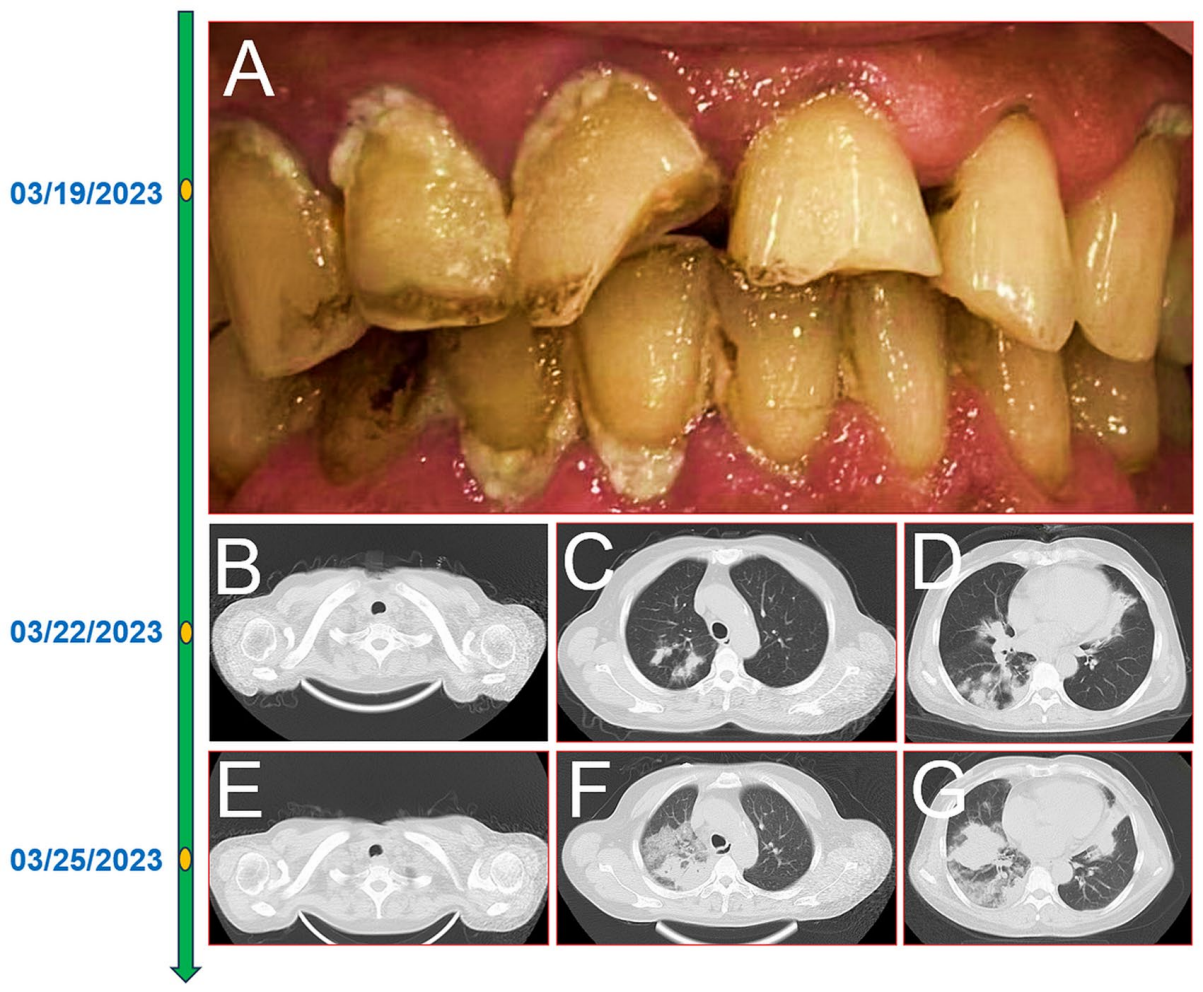
The timeline of the patient’ disease progression. Gingivitis and oral hygiene of the patients **(A)**; 16-slice computerized tomography images of the chest at initial diagnosis of the patient, neck **(B)**, upper lobe **(C)**, lower lobe **(D)**; 16-slice computerized tomography images of the chest in the advanced stage of the patient, neck **(E)**, upper lobe **(F)**, lower lobe **(G)**.

**Figure 2:**
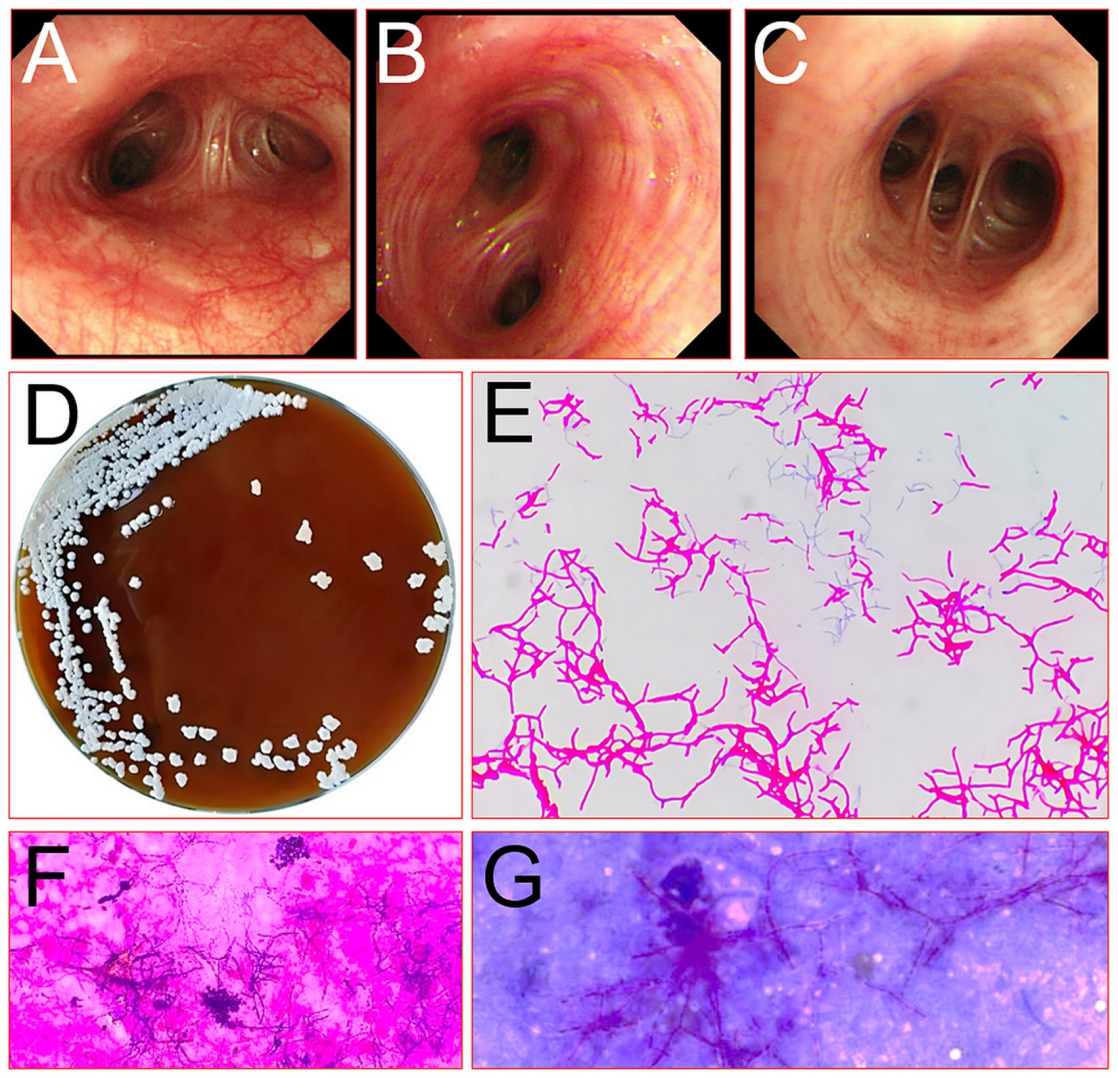
Picture of the bronchus of the right lung of the patient under a fiberoptic bronchoscope (**A**, main bronchus; **B**, lower lobe bronchus; **C**, basal segment bronchus) and microbe detected by fiberoptic bronchoscopic lavage fluid smear (**D**, culture original; **E**, micrograph of pathogenic bacteria; **F**, the standard acid-fast stain; **G**, the modified weakly acid-fast).

**Figure 3:**
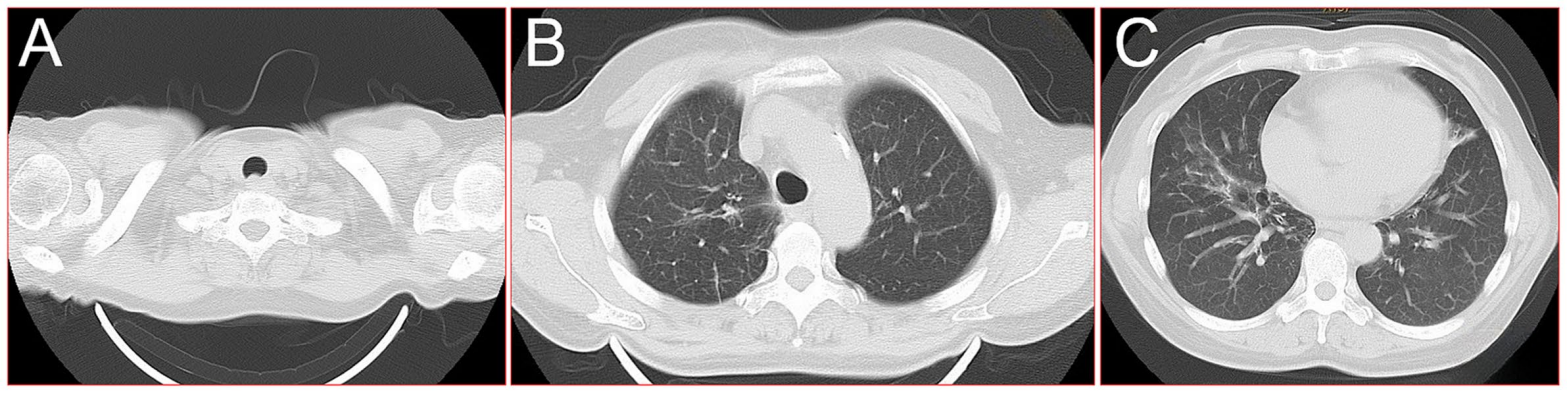
16-slice computerized tomography images of the chest during follow-up after the patient was cured. **(A)** Neck; **(B)** upper lobe; **(C)** lower lobe.

A 60-year-old female farmer, previously healthy, with no immune-related diseases. The patient visited the hospital due to gingival pain and pharyngeal discomfort. The body temperature was 38.6°C, the gums were red and swollen (Figure 1A), the pharynx was congested, and the tonsils were bilateral II degree enlarged. A diagnosis of tonsillitis and gingivitis was made by the community doctor. She was treated with dental cleaning and oral metronidazole. However, the symptoms did not relieve. Three days later, the patient developed dyspnea. Physical examination revealed tachypnea (42 beats/min), moist rales in both lungs, low breath sounds in the right lower lung, a regular heart rate of 131 beats /min, and no obvious heart murmur. The abdomen was soft and non-tender. The patient underwent chest CT, which revealed multiple solid opacities in the right lung (Figures 1B–D). The radiologist believes that pulmonary tuberculosis should be the preferred consideration. The patient’s hemocyte, blood biochemical, and blood tuberculosis laboratory tests are shown in Table 1. After multidisciplinary team (MDT) discussion based on the medical history, the diagnosis of this case should be considered pulmonary infection firstly, and the possibility of tuberculosis cannot be excluded. Therefore, the patient was treated with intravenous ceftriaxone sodium, an intravenous anti-inflammatory therapy. After 3 days of treatment, the patient’s symptoms worsened tachypnea developed. A repeat CT scan of the chest was performed, which revealed a markedly enlarged area of patchy hyperdensity in the right lung and spread to the left lung (Figures 1E–G). In cases where respiration could not be maintained, the patient was placed a ventilator assisted respiration with endotracheal intubation. Blood and sputum cultures were obtained, and both were negative. After MDT discussion again, G test, GM test and cryptococcus detection should be improved, and fiberoptic bronchoscopy should be improved if conditions permit. Pulmonary fungal infection combined with bacterial infection should be considered in the diagnosis. Intravenous meropenem combined with voriconazole was given as anti-inflammatory treatment. Subsequent tests, G test, GM test, cryptococcal antigen were all negative. No abnormalities were found in the bronchi except for thick sputum. No tumor or severe inflammation was found in the trachea by fiberoptic bronchoscopy (Figures 2A–C). As an unexpected bonus, Nocardia was found the bronchoalveolar lavage fluid smear and a week of culture (Figures 2D–G). At this point, the patient was diagnosed multisite infection by Nocardia. Based on the drug susceptibility analysis of bacterial culture, compound sulfamethoxazole 1.5 g every 6 h was given nasal feeding combined with intravenous meropenem. Two days later, the patient’s temperature got normalized, her spirits got improved gradually. The symptoms of gingival pain, pharyngeal discomfort and dyspnea with her did relieve also. Two weeks after treatment, the patient underwent a repeat CT scan of the chest, which revealed a reduction in the extent of bilateral lung shadow. The patient was scheduled to be discharged with oral trimethoprim-sulfamethoxazole for another 3 months. Subsequent follow-up chest CT showed that the lesions in both lungs were basically absorbed (Figures 3A–C). The symptoms of the oral and respiratory with her completely disappeared, and she was very satisfied with the treatment.

**Table 1:** Laboratory test results of the patient.

Laboratory tests	Normal range	Results
WBC (×109/L)	4 ~ 10	15.31
Neutrophile granulocyte percentage (%) Eosinophils percentage (%) Monocytes percentage (%)	50 ~ 70 0 ~ 5 3 ~ 8	90.6 0.5 2.1
C-reactive protein (mg/L) Erythrocyte sedimentation rate (mm/h)	<22 0 ~ 10	203 22
Albumin (g/L)	0 ~ 40	31.3
Alanine aminotransferase (U/L)	7 ~ 40	31
Aspartate aminotransferase (U/L)	13 ~ 35	21
Direct bilirubin (μmol/L)	0 ~ 6.8	4.2
Creatinine (μmol/L)	41 ~ 81	46.2
Urea nitrogen (μmol/L)	3.6 ~ 9.5	5.11
Procalcitonin (ng/mL) Interleukin-6 (pg/mL) *Mycoplasma pneumoniae* antibody *Chlamydia pneumoniae* antibody T-SPOT-TB	<0.05 0 ~ 40 Negative Negative Negative	90.36 >5,000 Negative Negative Positive
Protein (Urinalysis routine)	Negative	0
Urobilinogen (Urinalysis routine)	Negative	0
Occult blood (Urinalysis routine)	Negative	0

## Discussion

Nocardia is an aerobic prokaryotic actinomycete, widely exists in soil, air, decaying plants and other organic matter (8). It is an opportunistic pathogen of human, livestock and poultry. So far, a total of 791 nocardia isolates have been identified, of which 119 Nocardia species with effective names have been confirmed in literature, and 54 Nocardia species are related to human infection (9). It does not belong to the normal flora of human body, so it is not an endogenous infection. So, if this bacterium is detected in laboratory culture or NGS, it must be pathogenic. Unlike Candida, which can colonize the human body (10). Nocardia species often invade the human body from respiratory tract, oral mucosa lesions and skin lesions, and spread to lung, brain and other organs through respiratory tract and blood, which is easy to cause infection (11). Nocardial is aerobic, and the colonies were smooth and moist when cultured at 37° with general medium, grows into macroscopic colonies within 2–6 days, obtain satisfactory culture results requiring 4–6 weeks (12, 13). The colonies varied in color, including cheese, yellow, pink, coral red, and orange red (14, 15).

Besides, Nocardia belongs to the order actinomycetes, which was first isolated by Nocard in 1888 (16). It is similar to that of *Mycobacterium tuberculosis* (17). However, Nocardial mycelial ends did not show club-like expansion, different concentrations of decolorization solution were used for acid-fast staining, and the lower the concentration of decolorization solution used, the higher the positive rate of acid-fast staining (18). *Mycobacterium tuberculosis* has strong acid resistance and is not easy to decolorize. Therefore, the weakly acid-fast staining method can be used to distinguish Nocardia from Mycobacteria.

Nocardia pneumonia should be differentiated from actinomyces pneumonia and aspergillus pneumonia in chest imaging (19). Sulfur particles can be found in actinomyces pneumonia, and aspergillus pneumonia is the most common pulmonary fungal disease (20). The typical clinical manifestations of Nocardia pneumonia in the early stage were nodules or masses with halo sign around them, and crescent sign could be seen when cavitation was formed (20). However, their imaging identification is complicated and difficult in clinical practice (21).

These specific factors determine the complexity of the diagnosis of Nocardia, which is why the patient in this case was not diagnosed for days. It is difficult to culture Nocardia from sputum, for one thing, it is difficult to detect by smear staining, which is often misinterpreted by other bacteria and causes false positive results (15). On the other hand, it is because of the rapid growth of oral flora during sputum culture, which often leads to the suppression of nocardia. In addition, the commonly used media and culture conditions are not conducive to the cultivation of Nocardia, which will also lead to false negative results. In addition, the growth of Nocardia is slow, and the culture time is short in most laboratories, resulting in the missed detection of some nocardia. Nocardia is not the normal flora of the human body, so it is very important to detect the pathogen from tissue or body fluid secretions. Although the next generation sequencing (NGS) analysis testing of species, source, and drug sensitivity of pathogens has the advantages of fast speed and high sensitivity, the price is relatively expensive. In China, this testing has not been routinely carried out in hospital, can be shipped to commercial biological companies, so it is not very convenient. Bronchoalveolar lavage fluid obtained from fiberoptic bronchoscopy can obtain pathogenic bacteria in the lower respiratory tract, and it is rarely contaminated by other bacteria, which is a valuable sampling material for pathogenic bacteria in clinical work (22, 23).

Previous studies showed that nocardial pneumonia accounted for 85% of all nocardiosis cases and the in-hospital mortality rate was 15.0% (6). Delays in diagnosis and treatment are the most common reasons. The diagnosis and treatment of this case was also tortuous. She started with gums inflammation by the unclean mouth, and it is understandable that metronidazole was given to the dentist. Then, the inflammation rapidly progressed to the lungs. After ruling out mycoplasma and chlamydia pneumonia, the patient was treated empiric with ceftriaxone. However, Nocardia is sensitive to sulfonamides, aminoglycosides, some cephalosporins, carbapenems and quinolones, and sulfonamides are the first choice for treatment (20, 24). The advantages of this drug are its good oral bioavailability and its good permeability into tissues and cerebrospinal fluid. The dosage should be adequate and the course of treatment should be long. Six weeks for immunocompetent patients with localized pulmonary nocardiosis and at least 6 months for immunocompromised patients (20). For patients with central nervous system spread, this should be extended to 12 months. For people with AIDS, 12 months or more (25).

Besides, low-dose maintenance therapy is recommended for immunosuppressed patients. At present, due to the high drug resistance rate of sulfonamides, the total drug resistance rate is more than 40%, and the combination therapy is advocated (20, 25). Carbapenems and linezolid are the two drugs with high sensitivity. Linezolid is the first to be sensitive to almost all nocardial species and is indicated for severe infections, disseminated nocardiosis, and sulfonamides allergy. It is suggested that the above drugs can be preferentially selected according to the condition of the patient in the treatment of refractory pulmonary nocardiosis. If the diagnosis is delayed, the mortality rate may reach 30–50% (6, 20, 26, 27). Therefore, early and rapid diagnosis and treatment are of great significance for the prognosis of patients.

## Conclusion

This case illustrates the tortuous course of physician’s diagnosis and treatment of a healthy peasant woman with typical infection progression from oral infection to life-threatening pneumonia. From this tortuous process, we can learn the harmfulness of nocardiosis and the difficulty of diagnosis. It can provide some clinical thoughts for explaining a class of infectious diseases with common clinical symptoms but catastrophic outcomes, reduce medical disputes, and improve the diagnosis and treatment of rare diseases.

## Data availability statement

The original contributions presented in the study are included in the article/supplementary material, further inquiries can be directed to the corresponding authors.

## Ethics statement

Written informed consent was obtained from the individual(s) for the publication of any potentially identifiable images or data included in this article.

## Author contributions

KC: Data curation, Methodology, Project administration, Funding acquisition, Writing – review & editing. YW: Conceptualization, Formal analysis, Methodology, Resources, Software, Writing – original draft. JD: Data curation, Formal analysis, Investigation, Methodology, Software, Writing – original draft. P-SW: Conceptualization, Data curation, Formal analysis, Methodology, Writing – original draft. JY: Conceptualization, Data curation, Formal analysis, Funding acquisition, Investigation, Methodology, Project administration, Resources, Software, Supervision, Validation, Visualization, Writing – original draft, Writing – review & editing. G-PA: Conceptualization, Data curation, Formal analysis, Funding acquisition, Investigation, Methodology, Project administration, Resources, Software, Supervision, Validation, Visualization, Writing – review & editing.
